# Effect of Biometric Characteristics on the Change of Biomechanical Properties of the Human Cornea due to Cataract Surgery

**DOI:** 10.1155/2014/628019

**Published:** 2014-05-29

**Authors:** Xuefei Song, Achim Langenbucher, Zisis Gatzioufas, Berthold Seitz, Moatasem El-Husseiny

**Affiliations:** ^1^Department of Ophthalmology, Saarland University Medical Center, Kirrberger Straße 100, 66424 Homburg/Saar, Germany; ^2^Experimental Ophthalmology, Saarland University, Kirrberger Straße 100, 66424 Homburg/Saar, Germany

## Abstract

*Purpose.* To determine the impact of biometric characteristics on changes of biomechanical properties of the human cornea due to standard cataract surgery using biomechanical analysis. *Patients and Methods.* This prospective consecutive cross-sectional study comprised 54 eyes with cataract in stages I or II that underwent phacoemulsification and IOL implantation. CH, CRF, IOPg, and IOPcc intraocular pressure were measured by biomechanical analysis preoperatively and at 1 month postoperatively. Changes (Δ) were calculated as preoperative value versus postoperative value. Biometrical data were extracted from TMS-5 (CSI and SAI), IOLMaster (AL), and EM-3000 (CCT and ECC) preoperatively. *Results.* The average values of the changes were ΔCH = −0.45 ± 1.27 mmHg, ΔCRF = −0.88 ± 1.1 mmHg, ΔIOPg = −1.58 ± 3.15 mmHg, and ΔIOPcc = −1.45 ± 3.93 mmHg. The higher the CSI the smaller the decrease in CH (*r* = 0.302, *P* = 0.028). The higher the CCT the larger the decrease in CRF (*r* = −0.371, *P* = 0.013). The higher the AL the smaller the decrease in IOPg (*r* = 0.417, *P* = 0.005). The higher the AL, SAI, and EEC the smaller the decrease in IOPcc (*r* = 0.351, *P* = 0.001; *r* = −0.478, *P* < 0.001; *r* = 0.339, *P* = 0.013). *Conclusions.* Corneal biomechanical properties were affected by comprehensive factors after cataract surgery, including corneal endothelium properties, biometry, and geometrical characteristics.

## 1. Introduction

Refractive changes following cataract surgery were argued to be due to alterations of corneal biomechanical properties to a certain extent, in addition to implantation of the intraocular lens [1]. Ocular response analyzer (ORA; Reichert Inc., Depew, NY) is able to assess biomechanical properties of the cornea providing four variables the Goldmann-correlated IOP (IOPg), corneal-compensated IOP (IOPcc), corneal resistance factor (CRF), and corneal hysteresis (CH).

During an ORA examination, the response of the cornea due to a Gaussian shaped air puff is delayed due to hysteresis effects. The difference between these inward and outward motion applanation pressures is called corneal hysteresis, while the average of both provides IOPg [2]. According to the user's manual of ORA, it is believed that CRF is dominated by the elastic properties of the cornea and appears to be an indicator of the overall resistance of the cornea. IOPcc is a pressure measurement that utilizes the new information provided by CH to provide an IOP measure that is independent of or at least less affected by biomechanical properties of the cornea [3].

Reliability of the variables derived from ORA was studied before by different researchers [1, 4–9]. Relationship between ORA parameters and intraocular pressure (IOP) derived from various methods was also evaluated and discussed with differences and contradictions [3, 4, 10].

Corneal topography either with a Placido-based system or a Scheimpflug system is currently used in clinical routine for assessment of the corneal shape. Some statistical indices are extracted from these measurements which give a more detailed insight into the geometrical characteristics of the cornea: CSI represents center surround index and SAI refers to the surface asymmetry index, both of which were derived from TMS-5.

In biometrical examinations performed with IOLMaster, AL represents axial length in mm. A specular microscope is used for assessment of the properties of corneal endothelium. With that instrument, corneal central thickness (CCT in *μ*m) and endothelial cell density (ECC in cells per mm^2^) are extracted.

However, studies focusing on biomechanical properties and biometric characteristics are mostly pointing to single parameter or single aspect (topography, biometry, or endothelial cell measurement). Kamiya et al. concluded that CRF may reflect the overall rigidity of the cornea depending on CCT more correctly than CH does [11]. Alió et al. suggested that the change in CH was inversely correlated with AL, a finding that agrees with those in other studies of the relationship between CH value with a longer AL [12]. Focusing on relationship between geometrical characteristics and biomechanical properties, different researchers illustrated which in various ways [12, 13]. Furthermore, various factors (e.g., corneal edema, retained OVD, and compromised endothelium) can be present in the immediate postoperative period and play a role in the change in biomechanical parameters [1].

According to our knowledge, there are no various studies in the scientific literature about an effect of biometric characteristics on the change of biomechanical properties after standard phacoemulsification. We hypothesize that corneal biomechanical properties are not independent from the biometric characteristics. In other words, biomechanical properties are affected or overlaid by biometric characteristics.

The aim of this study is to synthetically investigate changes of biomechanical properties in terms of CH, CRF, IOPg, and IOPcc derived from the ORA measurement and to analyze potential influencing factors from corneal topography, biometry, and endothelial cell measurements.

## 2. Patients and Methods

### 2.1. Study Group and Protocol

This prospective consecutive cross-sectional study comprised 54 eyes with cataract in Stages I or II with visually significant lens opacification that underwent phacoemulsification (2.8 mm incision) from May 2012 to January 2013.

The study followed the tenets of the Declaration of Helsinki, and the local ethics committee of our university approved the study protocol. After receiving a thorough explanation of the procedure, risks, and potential complications of the study, all patients signed written informed consent forms.

Inclusion criteria were cataract eyes with an age between 40 and 80 years, no history of ocular surgery or corneal pathologies, and a normal fundus examination. Exclusion criteria were astigmatism of more than 3.00 diopters (D) or the need of any ocular surgery beside cataract extraction.

### 2.2. Patient Examination

All patients underwent a full ophthalmic examinations performed by the same experienced ophthalmologist (Xuefei Song), including slit lamp evaluation, inspection of the posterior segment of the eye, and visual acuity testing using the Snellen charts, tomography with Topographic Modeling System TMS-5 (TOMEY corp.), biometry with the IOLMaster (Carl Zeiss Meditec), corneal endothelium imaging performed with a specular microscope EM-3000 (TOMEY corp.), and biomechanical properties performed with the ORA (Reichert Inc., software version 3).

Together with all other ophthalmic examinations, indices gained from ORA were tested between 7 a.m. to 10 a.m., which largely avoided the diurnal fluctuation mentioned by the manufacture [2].

Standardized postoperative examinations were performed 4 weeks after cataract surgery to minimize the effect of wound healing on the biomechanics.

### 2.3. Surgical Technique

All phacoemulsification procedures were performed by the same surgeon (Moatasem El-Husseiny) using a retrobulbar anesthesia. A 2.8 mm clear corneal incision (CCI) was created using a corneal keratome at the steep meridian either temporally or superiorly. Two paracenteses less than 0.9 mm were performed 90 degree away from the main incision with the corneal keratome. Healon Ophthalmic Viscosurgical Device (OVD, Abbott Medical Optics, Illinois, USA) was injected in the anterior chamber and the capsulorhexis was performed using a capsulorhexis forceps. Hydrodissection and hydrodelineation were performed using balanced saline solution. Phacoemulsification (Alcon Infiniti, Alcon, Texas, USA) was achieved using the stop-and-chop technique. Bimanual irrigation and aspiration was used to remove the rest cortical matter. Healon was once again injected in the anterior chamber to facilitate the foldable intraocular lens (IOL) implantation. After that, Healon was aspirated bimanually. The incisions were hydrated and the wounds were tightly sealed. Postoperative topical ofloxacin 0.3% and prednisolone acetate 1% were used routinely.

### 2.4. Main Outcome Measures

All measures of the TMS-5, biometric characteristics of IOLMaster, endothelial cell measurement, and the ORA were recorded. As outcome measures, we restricted ourselves to those parameters, which showed a statistically significant effect on biomechanical properties change. CSI and SAI measured by TMS-5 were documented and calculated preoperatively; AL measured by IOLMaster was documented and calculated preoperatively; CCT and ECC measured by EM-3000 were documented and calculated preoperatively; CH, CRF, IOPg, and IOPcc measured by ORA were documented and calculated both preoperatively and postoperatively.

### 2.5. Statistical Analysis

The SPSS statistical software package for Windows (version 19.0, SPSS, Inc.) was used for statistical analysis. Data were expressed descriptively by mean ± standard deviation (SD), median, and range. In multiple linear regression analysis, Pearson's test (correlation coefficient *r*) was used to identify the potentially significant biometric parameters, which could affect the change of biomechanical properties due to cataract surgery. A *P* value less than 0.05 was considered statistically significant.

## 3. Results

In Table 1, the preoperative biometrical data from TMS-5, IOLMaster, and EM-300 are shown. Data making minor changes on biomechanical properties after cataract surgery are not shown, because no novel explanations could be derived.

Table 2 gives descriptive information for biomechanical property changes from the preoperative to the 1 month postoperative follow-up stage. Mean changes in the biomechanical measurements were ΔCH = −0.45 mmHg, ΔCRF = −0.88 mmHg, ΔIOPg = −1.58 mmHg, and ΔIOPcc = −0.35 mmHg.

Potential predictive biometric parameters for the change of biomechanical values (1 month preoperatively versus preoperatively) are shown in Table 3. The predictions are based on a multiple linear regression model, which is restricted to the relevant dependencies only. ΔCH, the average value of which was negative, showed a linear dependency on CSI, indicating that a large CSI value is affecting a smaller decrease of CH due to cataract surgery. Average value of ΔCRF was negative, which showed a linear dependency on CCT, indicating that a large CCT value refers to a larger decrease of CRF due to surgery. Mean value of ΔIOPg was negative, and ΔIOPg showed a linear dependency on AL, indicating that a large AL value refers to a smaller decrease of IOPg due to surgery. ΔIOPcc, whose average value was negative, showed a linear dependency on AL, SAI, and ECC, indicating that large AL, SAI, and ECC values are affecting a smaller decrease of IOPcc due to cataract surgery.

## 4. Discussion

Corneal hysteresis is related to the viscoelastic structure of corneal tissue, which can be attributed to the dampening effects of the cornea [12]. CSI is calculated from the difference in the average-area-corrected power between the central area (3.0 mm diameter) and an annulus surrounding the central area of the cornea. Together with Differential Sector Index (DSI) and Opposite Sector Index (OSI), CSI helps to differentiate between normal corneas (low value), regular astigmatism (low value), peripheral steepening keratoconus (low-middle value), and central steepening keratoconus (high value) [13]. Our study showed a positive correlation between CSI and the change of CH (regression formula: ΔCH = −0.695 + 1.28 · CSI). The mean value of ΔCH was negative, which indicates that a cornea with a higher power difference between center and surrounding may potentially indicate a smaller decline in viscous damping capacity of the corneal tissue due to cataract surgery, which is predominantly determined by the concentration and viscosity of the glycosaminoglycans and proteoglycans in corneal stroma, as well as by the collagen-matrix interactions [14]. Microscopic and immunohistochemical studies have shown significant misalignment of the stromal collagen lamellae in keratoconus, as well as abnormal distribution of collagen types and alterations of the proteoglycans in stromal extracellular matrix [15–17], which could partly explain these findings. With our data we could show that even in normal corneas the change of CH due to cataract surgery depends significantly on the preoperative value of the CSI index. However, further research focusing on microscopic morphological changes with those conditions needs to be performed more in detail.

We also investigated the factors influencing the change of CRF, which reflects the elastic properties and rigidity of the cornea [12]. Mean value of ΔCRF was negative and our regression analysis showed that the only influencing factor relevant to the change of CRF was the CCT (regression formula: ΔCRF = 6.072 − 0.013 · CCT). This indicated that a thicker cornea would potentially indicate a larger decline in total corneal resistance to deformation due to cataract surgery, resulting from the decrease of viscoelastic resistance of the cornea [18]. This result is in accordance with the literature results, which stated that a thicker cornea may potentially lead to a smaller change of CRF after standard cataract surgery [2, 3, 11]. This is due to the fact that CRF was originally calculated to maximize the correlation of CRF with CCT or to minimize the correlation of CRF with IOP, based on the manufacturer's large-scale clinical data analysis [2].

Focusing on the change of IOPg due to cataract surgery, we found a correlation with AL (regression formula: ΔIOPg = −21.134 + 0.854 · *AL*⁡). Interestingly, the cornea independent IOPcc was correlated with AL, SAI, and ECC (regression formula: ΔIOPcc = −25.373 + 0.734 · *AL*⁡+4.025 · SAI + 0.003 · ECC). Both average values of ΔIOPg and ΔIOPcc were negative. For both regressions (IOPg and IOPcc), axial length was positively correlated with IOPg and IOPcc, indicating that an eye with larger axial length may be potentially associated with a smaller decline in IOP due to cataract surgery. In accordance with our research, Kucumen et al. found that the mean IOPcc was statistically significantly lower at 3 months than preoperatively [1]. In contrast, according to research of Alió et al., myopic eyes with more extensibility and less resistance to the expansive force of normal IOP were accompanied with an increase in AL [12]. But the results of both studies are not comparable, because Alió et al. used a different population (myopic patient) compared to the present study (cataract patient). Findings made by Cho showed, especially in myopic eyes (with a large AL), that the change in IOP due to cataract surgery was significantly increased. Therefore, eyes with a large axial length may require more detailed monitoring of biomechanical properties and IOP postoperatively [19].

In general, a larger axial length is associated with a flatter geometry of the IOL, typically a more posterior position with the eye, and a lower refractive power.

SAI is a measure of central corneal asymmetry and is an important determinant of the optical performance of the anterior corneal surface [20], which allows for a prediction of spectacle-corrected visual acuity based on the corneal surface topography [21]. Resulting from our regression, a lower corneal surface asymmetry may potentially indicate a smaller decline in cornea compensated IOP due to cataract surgery.

A small fluctuation in IOP due to surgery may indicate satisfactory eye stability. ECC reflects the cell density in corneal endothelium. Moreover, a sufficient number is required for supporting dehydration of corneal stoma and maintenance of corneal clarity. These basic rules are supported by the results of our study that a sufficient endothelial cell count may potentially indicate smaller fluctuation in IOP after cataract surgery.

From this finding, we confirm the literature results that the cornea compensated IOP may offer an attractive alternative to traditional IOP measurement including Goldmann applanation tonometry. This might be of particular relevance for the evaluation and management of patients with normal IOP, where corneal biomechanics appear to play the greatest role for validity of IOP measurement [22]. However, absolute values of ΔIOPg were bigger than that of ΔIOPcc and both of these two values were negative, which indicates that IOPcc is less dependent on, but not independent of, corneal biomechanical properties.

Even if change in IOPg and IOPcc after one month of cataract surgery are detected to be statistically significant, they are not of significant clinical relevance. In contrast, changes of CRF and CH after the surgery as observed in our study may be of high clinical relevance.

We hypothesize that corneal biomechanical properties are not independent of the biometric characteristics. In other words, biomechanical properties are affected or overlaid by biometric characteristics. Up to our knowledge, this is the first study providing potential predictive biometric parameters for the change of biomechanical values due to cataract surgery. The roles of topography values, biometrical characteristics, and endothelial cell count on the change of biomechanical properties are discussed. ΔCH, ΔCRF, ΔIOPg, and ΔIOPcc decreased due to cataract surgery. If the predictive of changes in biomechanics are validated in larger study populations, they should be considered in the normal values provided by the manufacture of ORA. The limitation of this study was the sample size and limited assessed parameters.

We are aware that our prediction was formulated with the linear regression analysis as a result of this pilot study and based on a limited number of populations. They have to be updated, if a larger number of patients are analyzed. However, the general concept of predictively change of ORA values due to cataract surgery based on biomechanical measures is reasonable.

## Figures and Tables

**Table 1:** Descriptive information for biometric characteristics (preoperative).

	CSI [1]	SAI [1]	AL [mm]	CCT [*μ*m]	ECC [cells per mm^2^]
Mean ± SD	0.11 ± 0.43	0.53 ± 0.68	23.41 ± 1.25	546.08 ± 27.3	2428 ± 331
Median	0.12	0.34	23	542	2389
Min–max	−2.31–0.77	0.1–4.61	21.09–25.95	499–595	1364–3282

CSI = center surround index.

SAI = surface asymmetry index.

AL = axial length.

CCT = central cornea thickness.

ECC = endothelial cell count.

**Table 2:** Changes of biomechanical properties from preoperatively to 1 month postoperatively.

	ΔCH [mmHg]	ΔCRF [mmHg]	ΔIOPg [mmHg]	ΔIOPcc [mmHg]
Mean ± SD	−0.45 ± 1.27	−0.88 ± 1.1	−1.58 ± 3.15	−1.45 ± 3.93
Median	−0.5	−0.8	−1.4	−0.75
Min–max	−3.4–2.7	−3.1–1.7	−8.1–4.7	−13.1–6.6

CH = corneal hysteresis.

CRF = corneal resistance factor.

IOPg = Goldmann-correlated intraocular pressure.

IOPcc = corneal compensated intraocular pressure.

**Table 3:** Potential predictive biometric characteristics for the changes of biomechanical parameters (1 month postoperative versus preoperative).

Change of biomechanical parameters	Biometric characteristics					
	Constant for regression formula	CSI	SAI	AL	CCT	ECC
ΔCH	−0.695	1.28	—	—	—	—
*r* value	—	0.302	—	—	—	—
*P* value	0.001	0.028	—	—	—	—
ΔCRF	6.072	—	—	—	−0.013	—
*r* value	—	—	—	—	−0.371	—
*P* value	0.03	—	—	—	0.013	—
ΔIOPg	−21.134	—	—	0.854	—	—
*r* value	—	—	—	0.417	—	—
*P* value	0.004	—	—	0.005	—	—
ΔIOPcc	−25.373	—	4.025	0.734	—	0.003
*r* value	—	—	0.478	0.351	—	0.339
*P* value	0.003	—	<0.001	0.01	—	0.013

A“—” marks parameters, which did not affect biomechanical properties.

Regression coefficient in first row of each ORA parameter is representative with a linear regression function (e.g., ΔCH = −0.695 + 1.28 · CSI).

*r* value refers to correlation coefficient.

*P* value refers to significance level.

CSI = center surround index.

SAI = surface asymmetry index.

AL = axial length.

CCT = central cornea thickness.

ECC = endothelial cell count.

CH = corneal hysteresis.

CRF = corneal resistance factor.

IOPg = Goldmann-correlated intraocular pressure.

IOPcc = corneal compensated intraocular pressure.
